# Femoral nerve palsy caused by ileopectineal bursitis after total hip replacement: a case report

**DOI:** 10.1186/1752-1947-5-190

**Published:** 2011-05-18

**Authors:** Jan Liman, Philipp von Gottberg, Mathias Bähr, Pawel Kermer

**Affiliations:** 1 Department of Neurology, University of Göttingen, Robert-Koch-Str 40, 37075 Göttingen, Germany; 2 Department of Neuroradiology, University of Göttingen, Robert-Koch-Str 40, 37075 Göttingen, Germany

## Abstract

**Introduction:**

Infectious ileopectineal bursitis is a rare complication after total hip replacement and is associated mainly with rheumatoid arthritis. The main complications are local swelling and pain, but communication of the inflamed bursa with the joint can occur, leading to subsequent cartilage damage and bone destruction.

**Case presentation:**

We report a case of a 47-year-old Caucasian woman without rheumatoid arthritis who reported pain and palsy in her left leg almost one year after total hip replacement. She was diagnosed with an ileopectineal bursitis after total hip replacement, leading to femoral nerve palsy. The diagnosis was obtained by thorough clinical examination, the results of focused computed tomography and magnetic resonance imaging.

**Conclusion:**

To the best of our knowledge, this is the first report of non-infectious ileopectineal bursitis in a patient without rheumatoid arthritis as a complication of total hip replacement. This rare case underlines the importance of proper neurologic examination of persistent conditions after orthopedic intervention in otherwise healthy individuals. We believe this case should be useful for a broad spectrum of medical specialties, including orthopedics, neurology, radiology, and general practice.

## Introduction

Non-infectious ileopectineal bursitis is a rare complication after total hip replacement, associated mainly with rheumatoid arthritis (RA) or osteoarthritis [1-3]. The main complications are local swelling and pain, but communication of the inflamed bursa with the joint, with subsequent cartilage damage and bone destruction, can occur [4,5]. Only a few reports in the literature describe femoral neuropathies in patients with RA and iliopsoas bursitis [4,6,7].

## Case presentation

A 47-year-old Caucasian woman was referred to our department with a sub-acute paresis of her left leg, combined with a dull sensation in her inner thigh, knee, and lower leg. The paresis had increased over the past one-and-a-half weeks, and she was unable to walk freely without using walking aids on both sides.

Our patient reported that she had undergone replacement of her left hip eight months before presentation. During the first weeks after the intervention, she had experienced pain in her left buttock. On exploration, a hematoma had been discovered and treated accordingly. Within the following two months, she had again developed pain in the region of her left hip, attributed to loosening of the acetabular fossa, which was resolved by a second operation three months after the initial hip replacement. Our patient's recovery after the second operation was without incident. She was sent for rehabilitation, the strength in her left leg improved steadily, and she did not have pain.

Two weeks before presenting to our department, our patient suddenly noticed paresis during hip flexion, and to a lesser extent, during knee extension of her left leg. She consulted a neurologist, who referred her to our department after documentation of paresis of her left iliopsoas muscle and, to a lesser extent, her left quadriceps femoris muscle, accompanied by diminished patellar tendon reflexes on the affected side, and sensory loss in the supply area of the left femoral nerve. Magnetic resonance imaging (MRI) scans taken at that time showed an increased amount of fluid within her left ileopectineal bursa. Left-sided bursitis was diagnosed, without any explanation for the acute impairment of the femoral nerve.

At initial examination in our department, our patient reported pain in her left inguinal region. No local swelling or mass was palpable. A motor examination revealed normal strength in the arms, but a 3/5 paresis for hip flexion, and 4/5 paresis for knee extension on her left leg. The left patellar reflex was decreased; all other reflexes were symmetric and normal. A sensory examination showed paresthesia, with hypoesthesia in her left inner thigh, knee, and the inner part of her leg, corresponding to the area of supply for the femoral nerve.

Results of blood tests showed normal values for inflammation parameters, such as C-reactive protein, and white blood cell count.

**Figure 1:**
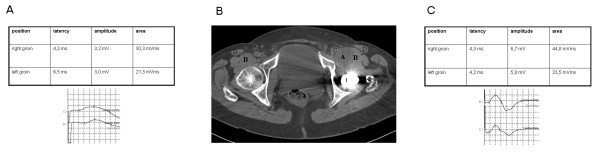
**Images of structures before and after treatment**. **(a) **Neurography of the femoral nerve before treatment showing prolonged latency of the left side. **(b) **CT image of the pelvis showing a cystic structure 4.8 × 3.7 mm in size, corresponding to (a) the ileopectineal bursa, medial to (b) the musculus iliopsoas, and dorsal to the (d) musculus sartorius, which caused a medial dislocation of (c) the femoral artery, vein, and femoral nerve. **(c) **Neurography of the femoral nerve four months after treatment, showing normal latency values.

Neurographic examination of her femoral nerve showed increased distal motor latency for her left femoral nerve on a side-to-side comparison (Figure 1a). No abnormalities were found in the tibial or sural nerves on either side. Electromyography showed some acute denervation, with rarefied maximal innervation of her left iliopsoas muscles (not shown). The findings were summarized as acute femoral nerve palsy.

A pelvic computed tomography (CT) scan with multi-planar reconstruction revealed a cystic structure, 4.8 × 3.7 cm in size, medial to the left inguinal fossa, and ventral to the left hip joint, corresponding to the ileopectineal bursa. This had caused a medial dislocation of the femoral artery, vein, and femoral nerve (Figure 1b).

The inflamed ileopectineal bursa was punctured, and about 10 ml of liquid was removed. Our patient reported a marked decrease in pain almost immediately afterward. Two days later, she was already able to walk without assistance for short distances.

We discharged our patient with a referral for physiotherapy, and she was told to see her general practitioner on a regular basis for sonographic control of the inflamed bursa. Two weeks later, she again presented with intense inguinal pain and increasing weakness of her left leg, and she was admitted to our department for a second time. We again found an increased bursa diameter, and we decided to perform orthopedic bursectomy of the left ileopectineal bursa. During surgery, the bursa was found to be about the size of a hen's egg, causing maximal stretching of the femoral nerve. Our patient's post-interventional course was free of complications, and she was discharged with a referral for rehabilitation. Three months later, she was seen at our out-patient clinic. She had only slight pain and subtle paresis of her left-sided hip flexion, and was able to walk even long distances without assistance. Neurography gave normal values for the femoral nerve on both sides (Figure 1c).

## Discussion

The ileopectineal bursa is located between the iliopsoas muscle, and the joint capsule, sometimes being connected to the joint itself. It is one of the largest bursae in the body, and the differential diagnosis of its inflammation includes femoral or inguinal hernia, lymphoma lymphadenopathy, psoas abscess and vascular abnormalities (for example, aneurysm). According to the literature, nerve injuries occur in 1% to 2% of patients who undergo total hip arthroplasty [8]. A palsy of the femoral nerve after hip arthroplasty is most commonly a result of hematoma within the iliacus muscle [9].

Only a few reports in the literature describe ileopectineal bursitis as a cause of femoral nerve palsy. In all cases reported in the literature, the patients either had underlying RA, which had previously caused a bursitis before hip replacement [10,11], or a degenerative joint disease underlying the bursitis [12].

Sub-acute infection of the implant after total hip replacement is another rare cause of bursitis that we considered in our patient; however, the bursa puncture and the intra-operative findings and blood samples did not show any signs of an infective cause for the inflammation.

Apart from femoral nerve lesions, another major complication of bursitis is opening of the bursa, and its communication with the joint, which may lead to progressive inflammation and, consecutively, destruction of the hip bone.

## Conclusion

Our case highlights a rare and, to the best of our knowledge, previously unreported but severe complication after routine surgery in a patient without a history of RA. It emphasizes the importance of post-surgical monitoring, especially if symptoms such as pain, numbness, or limb weakness develop or do not diminish. In our opinion, a neurologic examination investigating the possibility of a femoral nerve lesion should be performed early in the disease course. If suspicion exists of an inguinal mass being responsible for the nerve injury, then CT, MRI, and ultrasonography are reliable methods of investigation. They provide exact anatomic delimitation of the lesion and can easily distinguish between the fluid content of a cyst or, for example, lymphadenopathy or a hematoma in the muscle.

If an inflamed bursa is diagnosed as a cause of nerve palsy, the initial fluid amount can be relieved by puncturing the bursa. However if the pain recurs, and the bursa re-enlarges, it should be removed by surgical intervention early in the disease course.

## Consent

Written informed consent was obtained from the patient for publication of this case report and any accompanying images. A copy of the written consent is available for review by the Editor-in-Chief of this journal.

## Competing interests

The authors declare that they have no competing interests.

## Authors' contributions

JL examined the patient and was a major contributor to writing the manuscript. PG interpreted and prepared an MRI scan. MB interpreted the electrophysiological data and prepared the figure outline. PK examined the patient and was a major contributor to writing the manuscript. All authors read and approved the final manuscript.
